# GDF15 Contributes to Radioresistance by Mediating the EMT and Stemness of Breast Cancer Cells

**DOI:** 10.3390/ijms231810911

**Published:** 2022-09-18

**Authors:** Xinrui Zhao, Xinglong Liu, Songling Hu, Yan Pan, Jianghong Zhang, Guomei Tai, Chunlin Shao

**Affiliations:** 1 Institute of Radiation Medicine, Shanghai Medical College, Fudan University, Shanghai 200032, China; 2 Department of Radiotherapy, Nantong Tumor Hospital and the Affiliated Tumor Hospital of Nantong University, Nantong 226631, China

**Keywords:** bioinformatics analysis, GDF15, breast cancer cells, radioresistance, EMT, stem-like traits

## Abstract

Radiotherapy is one of the conventional methods for the clinical treatment of breast cancer. However, radioresistance has an adverse effect on the prognosis of breast cancer patients after radiotherapy. In this study, using bioinformatic analysis of GSE59732 and GSE59733 datasets in the Gene Expression Omnibus (GEO) database together with the prognosis database of breast cancer patients after radiotherapy, the *GDF15* gene was screened out to be related to the poor prognosis of breast cancer after radiotherapy. Compared with radiosensitive parental breast cancer cells, breast cancer cells with acquired radioresistance exhibited a high level of GDF15 expression and enhanced epithelial-to-mesenchymal transition (EMT) properties of migration and invasion, as well as obvious stem-like traits, including the increases of mammosphere formation ability, the proportion of stem cells (CD44^+^ CD24^−^ cells), and the expressions of stem cell-related markers (SOX2, NANOG). Moreover, knockdown of *GDF15* sensitized the radioresistance cells to irradiation and significantly inhibited their EMT and stem-like traits, indicating that GDF15 promoted the radioresistance of breast cancer by enhancing the properties of EMT and stemness. Conclusively, GDF15 may be applicable as a novel prognosis-related biomarker and a potential therapeutic target for breast cancer radiotherapy.

## 1. Introduction

Breast cancer is one of the most common malignancies worldwide, and it ranks as the second leading cause of cancer-related death in women [1,2]. Surgical treatment, radiotherapy and chemotherapy are the pivotal and effective therapeutic approaches for breast cancer [3,4]. Although the effect of radiotherapy was remarkable, after 10 years of adjuvant radiotherapy following breast conserving surgery, the recurrence and mortality rates were 15.7% and 3.8%, respectively [5], where radioresistance of breast cancer led to adverse outcomes in a majority of patients [6,7]. This recurrence jeopardizes the life of breast cancer patients, along with a higher risk of metastasis and poor prognosis [8,9]. Hence, identifying the key biomarkers and understanding the mechanism of breast cancer recurrence may promote novel diagnostic and therapeutic strategies to ameliorate the prognosis of breast cancer.

Epithelial-to-mesenchymal transition (EMT) is a cell trans-differentiation progress in which epithelial cells acquire mesenchymal characteristics [10]. In recent years, EMT has been considered an important step in the invasion and metastasis of many kinds of cancers [11]. It was reported that stress-induced EMT contributed to radioresistance [12,13]. In addition, cancer stem-like cells (CSCs) are a unique subset of cells that possess a distinctive ability to perpetuate the growth of malignant cells infinitely [14], and CSCs are closely related to tumorigenesis, treatment-resistance and metastasis [15,16]. Due to the ability of self-renewal and repopulation, CSCs impede the efficacy of clinical radiotherapy (RT) [17]. Clinicopathological studies have demonstrated that a high frequency of CSCs in the solid tumor is related to low tumor shrinkage, leading to the poor outcome of different types of tumors, including glioblastoma, head and neck squamous cell carcinoma, breast cancer and rectal cancer [18,19]. Accumulating studies reveal that cells undergoing EMT may exhibit CSC traits and play an important role in the cells with CSC properties [20,21]. Therefore, it is of great biological and clinical significance to explore the mechanism and find new biomarkers of radioresistant breast cancer for enhancing the efficiency of RT.

Growth differentiation factor 15 (GDF-15), a 25 kDa homodimer, classified as a member of the transforming growth factor beta (TGF-β) superfamily and a secreted protein circulating in plasma, was first identified as a novel transcript from a macrophage cell line in 1997. It is also known as macrophage inhibitory cytokine-1 (MIC-1) [22,23,24]. It was reported that GDF15 contributed to multiple pathological processes, including inflammation, cancer, cardiovascular diseases, and obesity [23,25], and it could be applied as a biomarker of radioresistance of human fibroblast cells, oral cancer and lung cancer [26,27,28]. Nevertheless, to date, it is still unclear whether GDF15 is relative to the radioresistance of breast cancer.

In the study, with a bioinformatics analysis of the GEO database and the survival database of breast cancer patients, we screened out the potential target molecule GDF15 associated with poor prognosis of breast cancer patients after radiotherapy. Further investigation revealed that GDF15 could mediate the radioresistance of breast cancer cells by promoting the EMT properties and stem-like traits. These findings proposed a novel therapeutic target for the clinical treatment of radioresistant breast cancer.

## 2. Results

### 2.1. Identification of Differential Expressed Genes (DEGs) in the Breast Cancer Lines of GSE59732 Dataset

**Figure 1:**
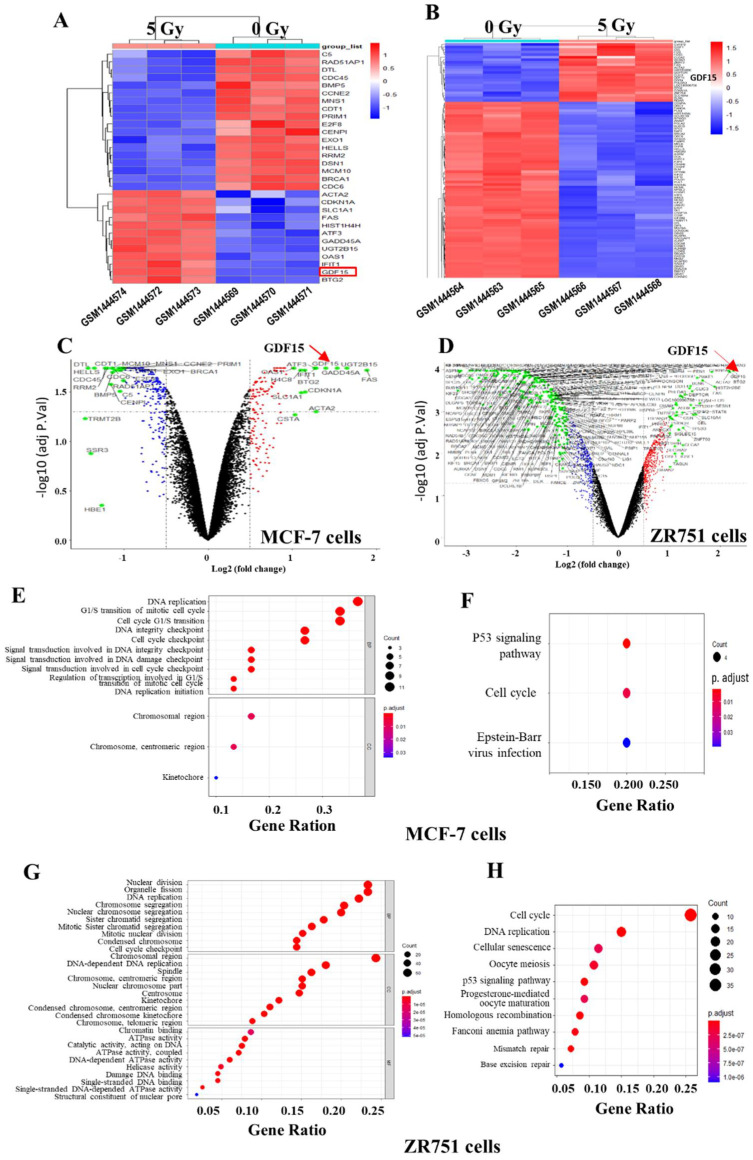
Identification of DEGs in the unirradiated (0 Gy) and irradiated (5 Gy) breast cancer cell lines in the GSE59732 dataset. (**A**,**B**) Heatmaps show 30 DEGs in MCF-7 cells (**A**) and 280 DEGs in ZR751 cells (**B**). Red and blue panes indicate the up-regulated and down-regulated genes, respectively. (**C**,**D**) Volcano plots of above 30 DEGs in MCF-7 cells (**C**) and 280 DEGs in ZR751 cells (**D**). Red and green dots represent the up-regulated and down-regulated genes, respectively. *p* < 0.05 and |log 2(FC)| > 1 are set as the threshold. (**E**,**G**) GO analysis of the components of biological process (BP), cellular component (CC) and molecular function (MF) of the DEGs in MCF-7 cells (**E**) and ZR751 cells (**G**). (**F**,**H**) KEGG analysis of the enriched pathways of DEGs in MCF-7 cells (**F**) and ZR751 cells (**H**).

We firstly analyzed the microarray information of dataset GSE59732 acquired from the GEO database and identified 30 DEGs, including 12 up-regulated genes and 18 down-regulated genes in MCF-7 cells (GSM1444569–GSM1444574), and 280 DEGs, including 43 up-regulated genes and 237 down-regulated genes in ZR751 cells (GSM1444563–GSM1444568), the DEGs screening is based on the ratio of changes in gene expression in unirradiated (0 Gy) and irradiated (5 Gy) cells. (Figure 1A,B). The volcano plots illustrated the distribution of the DGEs (bright green dots) between 0 and 5 Gy irradiated cells of above two cell lines (*p* < 0.05 and |log2Fold Change (FC)| > 1) (Figure 1C,D). To know DEGs-related signaling pathways, we performed GO (Gene Ontology) and KEGG (Kyoto Encyclopedia of Genes and Genomes) analyses and found that, for MCF-7 cells, the DEGs were mainly enriched in the regulation of DNA replication, cell cycle process, and chromosomal region for cellular components (Figure 1E). With the KEGG pathway evaluation, we clearly elicited two enriched signaling pathways associated with the p53 signaling pathway and cell cycle pathway (Figure 1F). For ZR751 cells, the DEGs were concentrated in the biological process of nuclear division, the DNA replication and mitosis process, the chromosomal region for cellular components, and catalytic activity on DNA related to molecular function (Figure 1G). The KEGG pathway analysis showed that the DEGs of ZR751 cells were also chiefly enriched in the p53 signaling pathway and cell cycle pathway (Figure 1H). The top 10 up-regulated DEGs and down-regulated DEGs in the irradiated MCF-7 and ZR751 cells are listed in Tables S1 and S2, respectively.

### 2.2. Identification of DEGs in the Breast Cancer of GSE59733 Dataset and the Network Analysis of the Overlapping DEGs between GSE59732 and GSE59733 Dataset

**Figure 2:**
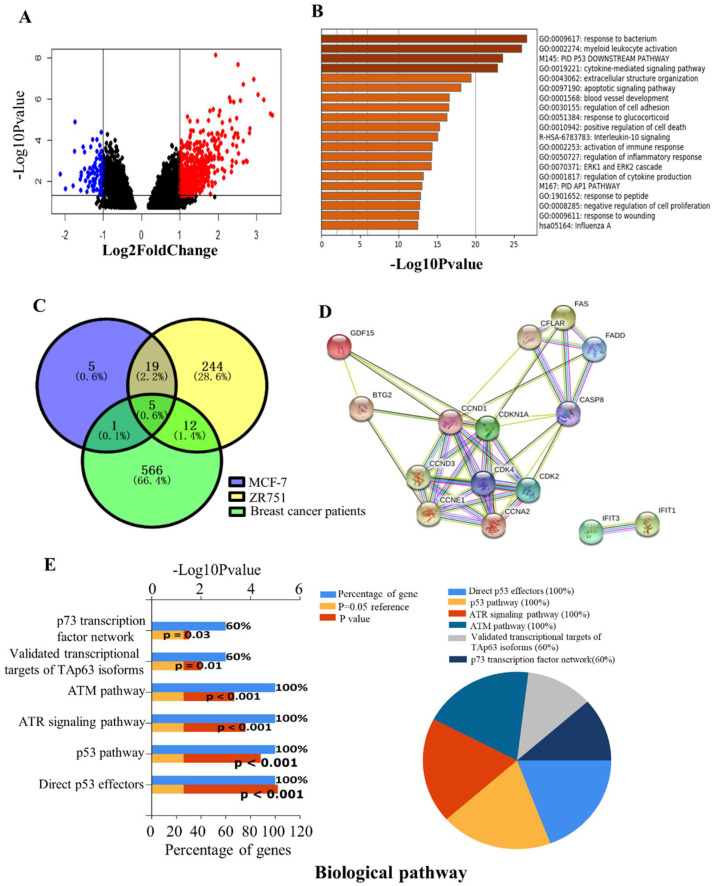
Identification of DEGs in the breast cancer patients of the GSE59733 dataset and the functional analysis of the overlapping DEGs between the GSE59732 dataset and GSE59733 dataset. (**A**) Volcano plots showing 585 DEGs in the tumor samples of breast cancer patients pre- and post- radiotherapy. Red and blue dots represent the up-regulated and down-regulated genes based on an adjusted *p* < 0.05 and |log 2(FC)| > 1. Black dots: no significant change. (**B**) Metascape analysis of the enriched pathways of above 585 DEGs. (**C**) Venn diagrams of DEGs in MCF-7 and ZR751 cell lines from GSE59732 dataset and breast cancer patients from GSE59733 dataset. A total of 5 intersecting genes (GDF15, IFIT1, CDKN1A, FAS, BTG2) were screened out and were all up-regulated. (**D**) The PPI network of these 5 DEGs and their related genes, created by the online tool STRING. (**E**) The biological pathways of DEGs were analyzed by the FunRich and showed as a bar chart (**left**) and pie chart (**right**), respectively. In the bar chart, the upper *x*-axis represents the *p*-value (−log10), and the lower *x*-axis represents the percentage of genes (blue). The *y*-axis represents the GO terms. Yellow represents the reference *p*-value 0.05, and red represents the specific *p*-value. The longer the rectangular zone, the smaller the *p*-value is. In the pie chart, each color represents a signaling pathway, and the proportion of DEGs participating in each signaling pathway in the 5 total DEGS showed in the form of the percentage.

Between the paired tumor samples pre-radiotherapy (GSM1444655–GSM1444657, GSM1444659–GSM1444664) and post-radiotherapy (GSM1444653, GSM1444654, GSM1444658, GSM1444666, GSM1444668–GSM1444671) in GSE59733 dataset, a total of 585 DEGs, including 500 up-regulated genes and 85 down-regulated genes (*p* < 0.05 and |log2 Fold  Change  (FC)| > 1), were screened out with the GEO2R tool. Volcano map revealed the clusters of DEGs after batch correction, where red and green dots indicated the up-regulated and down-regulated genes, respectively (Figure 2A). After analyzing these hundreds of DEGs with R language, several major high-score pathways were enriched (Figure 2B). The top 10 up-regulated DEGs and down-regulated DEGs acquired from the analysis of the paired tumor samples before and after radiotherapy were displayed in Table S3.

Given the above analysis in the GSE59732 and GSE59733 dataset, five intersecting DEGs (GDF15, IFIT1, CDKN1A, FAS, BTG2) were screened out among 30 DEGs in the MCF-7 cell line, 280 DEGs in the ZR751 cell line and 585 DEGs in tumor samples of breast cancer patients (Figure 2C). Compared with the non-irradiated samples, all these five genes were up-regulated in the irradiated MCF-7, ZR751 cells and the tumor of breast cancer patients after radiotherapy. The PPI network analysis of these DEGs using the online tool STRING predicts that GDF15, BTG2, CDKN1A and FAS are connected with a core of CDKN1A, but IFIT1 is isolated (Figure 2D). To further explore the biological function of these five DEGs, we conducted a biological pathway enrichment analysis using FunRich software 3.1.3 (open access). The result showed that among six significantly enriched pathways, p53, ATR and ATM signaling pathways were particularly important to radiation responses (Figure 2E).

### 2.3. Expression of DEGs in the Clinical Samples of Breast Cancer

**Figure 3:**
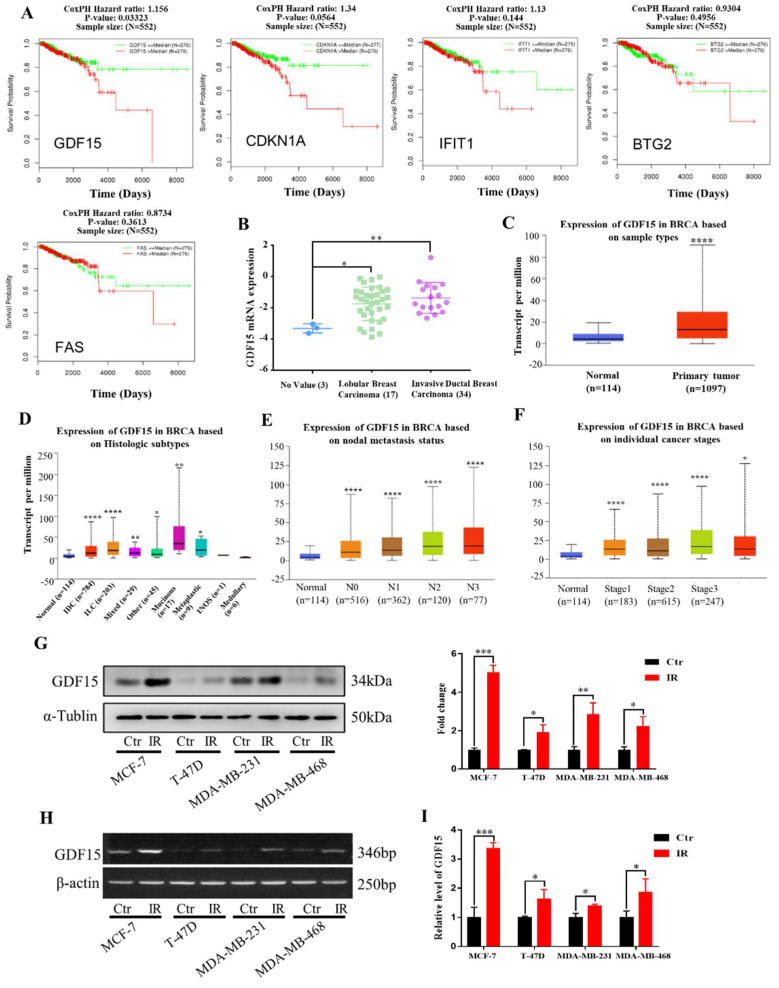
Expressions of 5 DEGs in the clinical samples of breast cancer patients and the GDF15 expressions in different breast cancer cells. (**A**) Relationship of the expression levels of DEGs (GDF15, IFIT1, CDKN1A, FAS, BTG2) and the survival of 552 breast cancer patients in the LinkedOmics database. (**B**) The mRNA level of GDF15 in lobular breast carcinoma and invasive ductal breast carcinoma was higher than that in normal tissues. Data were obtained from Oncomine database. (**C**) GDF15 mRNA was highly up-regulated in breast cancer samples in comparison with normal breast samples. (**D**) The expression of GDF15 in different histologic subtypes of breast cancer was higher than that in normal breast tissue. (**E**,**F**) The elevated GDF15 was significantly correlated with nodal metastasis status and individual cancer stages of breast cancer. (**G**) Western blot assay of the GDF15 protein expression in MCF-7, T-47D, MDA-MB-231 and MDA-MB-468 cells irradiated with 5 Gy X-rays or sham-irradiated. (**H**,**I**) RT-PCR and qRT-PCR assay of GDF15 mRNA expression in above cells. **** *p* < 0.0001, *** *p* < 0.001, ** *p* < 0.01, * *p* < 0.05.

To verify the correlation between DEGs and clinical outcome, by means of the LinkedOmics database, we analyzed the relationship of the above five DEGs with the survival of 552 patients after radiotherapy and found that only the low expression of GDF15 was statistically coordinated with the overall survival of breast cancer patients after radiotherapy (*p* = 0.03) (Figure 3A). We further validated the relationship between GDF15 and the clinical profile of breast cancer patients by employing the Oncomine database and BRCA (breast invasive carcinoma) dataset of the TCGA database. The clinical tissue samples of breast cancer collected in the Zhao breast dataset of the Oncomine database showed that the mRNA expressions of GDF15 in both lobular breast carcinoma and invasive ductal breast carcinoma were higher than that in normal tissue (Figure 3B). The TCGA database also revealed the relationship between the expression level of GDF15 and the clinicopathological characteristics of breast cancer patients. In comparison with the breast tissue specimens of 114 normal healthy individuals, the expression of GDF15 was significantly higher in 1097 breast cancer samples (*p* < 0.0001) (Figure 3C), including 784 infiltrating ductal carcinomas (IDC) (*p* < 0.0001), 203 infiltrating lobular carcinomas (ILC) (*p* < 0.0001), 29 mixed histology (*p* < 0.01), 17 mucinous carcinomas (*p* < 0.01), and 9 metaplastic carcinomas (*p* < 0.05) (Figure 3D). Consistently, the elevated GDF15 expression was also positively correlated with the patient’s nodal metastasis and individual cancer stages (Figure 3E,F). These data indicate that GDF15 may be involved in the development of breast cancer.

According to the above bioinformatics analyses, we subsequently verified radiation-induced alterations of GDF15 in human breast cancer cell lines (MCF-7, T-47D, MDA-MB-23, MDA-MB-468). Judging from the Western blot assay, the expression of GDF15 was obviously increased in the MCF-7, T-47D, MDA-MB-231 and MDA-MB-468 cell lines after five Gy X-ray irradiations (Figure 3G). Consistent with above results, after irradiation, the mRNA expression levels of GDF15 were also up-regulated in these breast cancer cell lines based on the RT-PCR and qRT-PCR assay, respectively (Figure 3H,I).

### 2.4. Enhancement of EMT and CSCs Properties in the Radioresistant Breast Cancer Cells

**Figure 4:**
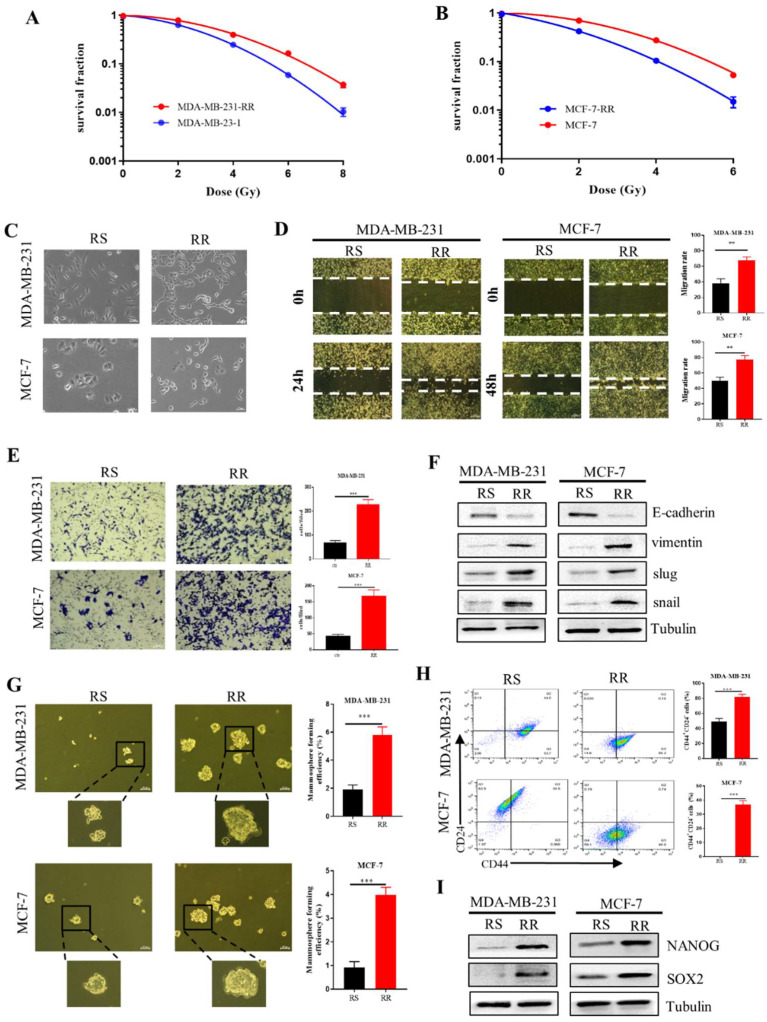
The enhanced EMT and stem-like traits in radioresistant breast cancer cells. (**A**,**B**) Clonogenic survivals of radiosensitive MDA-MB-231 and MCF-7 cells and radioresistant MDA-MB-231RR and MCF-7RR cells after irradiation. (**C**) The representative morphologies of radiosensitive (RS) and radioresistant (RR) MDA-MB-231 and MCF-7 cells. Scale bars, 50 μm. (**D**) Wound healing assays of the migration rates of indicated breast cancer cells. Scale bars, 250 μm. (**E**) Transwell assays were employed to examine the invasion of indicated breast cancer cells. Scale bars, 100 μm. (**F**) The protein expression of snail, slug, vimentin and E-cadherin related to the EMT process was detected by Western Blot. (**G**) Mammosphere formation assay was conducted to test the stemness of indicated breast cancer cells. Spheres bigger than 50 μm were quantified. Scale bars, 100 μm. (**H**) The percentages of CD44^+^ CD24^−^ cells in the indicated breast cancer cells detected by flow cytometry. (**I**) Western blot assay of the expressions of stemness-related markers of NANOG and SOX2 in the indicated breast cancer cells. ** *p* < 0.01, *** *p* < 0.001.

We established two radioresistant (RR) cell lines by periodically irradiating human breast cancer cells MDA-MB-231 and MCF-7 to X-rays and named these RR cell lines as MDA-MB-231-RR and MCF-7-RR, respectively. The radioresistance of RR cells was confirmed by clonogenic survival assay (Figure 4A,B). Considering the role of EMT in the promotion of cancer radioresistance [29,30], we observed the morphological changes of radioresistant cells and found that MDA-MB-231-RR and MCF-7-RR cells appeared to be a fibroblast-like phenotype with elongated and more leading-edge protrusions (Figure 4C). Meanwhile, these RR cells had stronger abilities of wound healing and invasion (Figure 4D,E), together with higher protein expression levels of snail, slug and vimentin and lower expression of E-cadherin in comparison with their radiosensitive parental cells, demonstrating that the radioresistant breast cancer cells possessed stronger EMT properties.

EMT is also involved in the generation of CSCs [31], which limits the efficacy of radiotherapy [16]. We found that the ability of the mammosphere formation ability in MDA-MB-231-RR and MCF-7-RR cells was significantly higher than that of their parental cell lines (Figure 4G), indicating that the radioresistant cells possessed stem cell-like traits. Further investigation demonstrated that more CD44^+^ CD24^−^ cells were enriched in the radioresistant cells (Figure 4H). As the core transcriptional markers in maintaining the CSC population, NANOG and SOX2 were also highly expressed in the radioresistant cells (Figure 4I). These findings collectively demonstrated that the radioresistant breast cancer cells exhibited EMT characters and had stem-like traits.

### 2.5. Inhibition of GDF15 Attenuates the EMT Properties of Radioresistant Breast Cancer Cells

**Figure 5:**
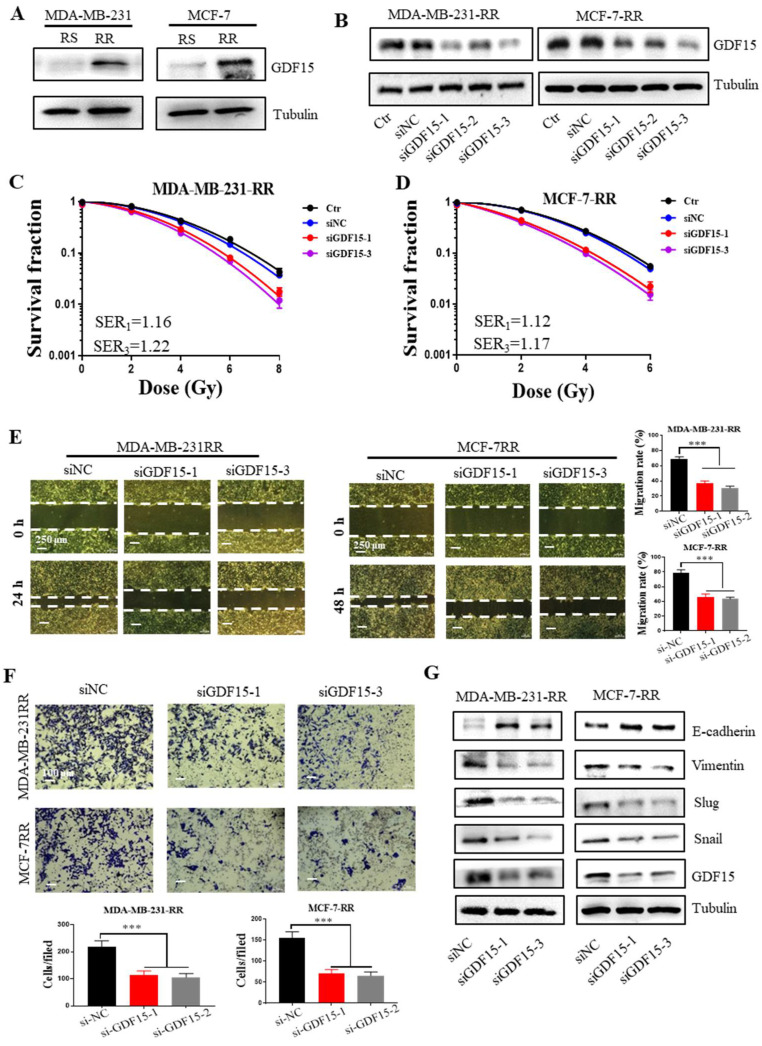
GDF15 knockdown impaired the EMT features of MDA-MB-231-RR and MCF-7-RR cells. (**A**) Western blot assay of the protein expression of GDF15 in the radiosensitive (RS) and radioresistant (RR) MDA-MB-231 and MCF-7 cells. (**B**) Western blot assay of GDF15 in MDA-MB-231RR and MCF-7RR cells transferred with different siGDF15 for 48 h. The most effective siGDF15-1 and siGDF15-3 were used for further GDF15 silence treatment. (**C**,**D**) Influence of siGDF15 on the survival of radioresistant cells. (**E**) Wound healing assay of the influence of siGDF15 on the ability of migration of radioresistant cells. (**F**) Transwell assay of the influence of siGDF15 on the ability of invasion of radioresistant cells. (**G**) Western blot assay of EMT-associated makers (snail, slug, vimentin, E-cadherin) in radioresistant cells transfected with siGDF15. *** *p* < 0.001.

Next, we want to know whether GDF15 plays an essential role in promoting the EMT process of radioresistant breast cancer cells. Western bolt assay showed that the expression levels of GDF15 in radioresistant MDA-MB-231-RR and MCF-7-RR cells were much higher than those in their radiosensitive (RS) parental cells (Figure 5A). When these radioresistant cells were transfected with GDF15-targeting siRNA (si-GDF15) to suppress the expression of GDF15 (Figure 5B), their clonogenic survivals were significantly reduced in comparison with the siNC group after irradiation, indicating that the knockdown of GDF15 efficiently enhanced the radiosensitivity (Figure 5C,D). In addition, the high migration and invasion abilities of the radioresistant cells were significantly weakened after knocking-down GDF15 (Figure 5E,F). Consistent with this, this siGDF15 transfection decreased the protein expressions of snail, slug, and vimentin and increased E-cadherin expression in the MDA-MB-231-RR and MCF-7-RR cells (Figure 5G). Accordingly, GDF15 promoted the EMT process of the radioresistant breast cancer cells.

### 2.6. Inhibition of GDF15 Decreases the Stemness of Radioresistant Breast Cancer Cells

**Figure 6:**
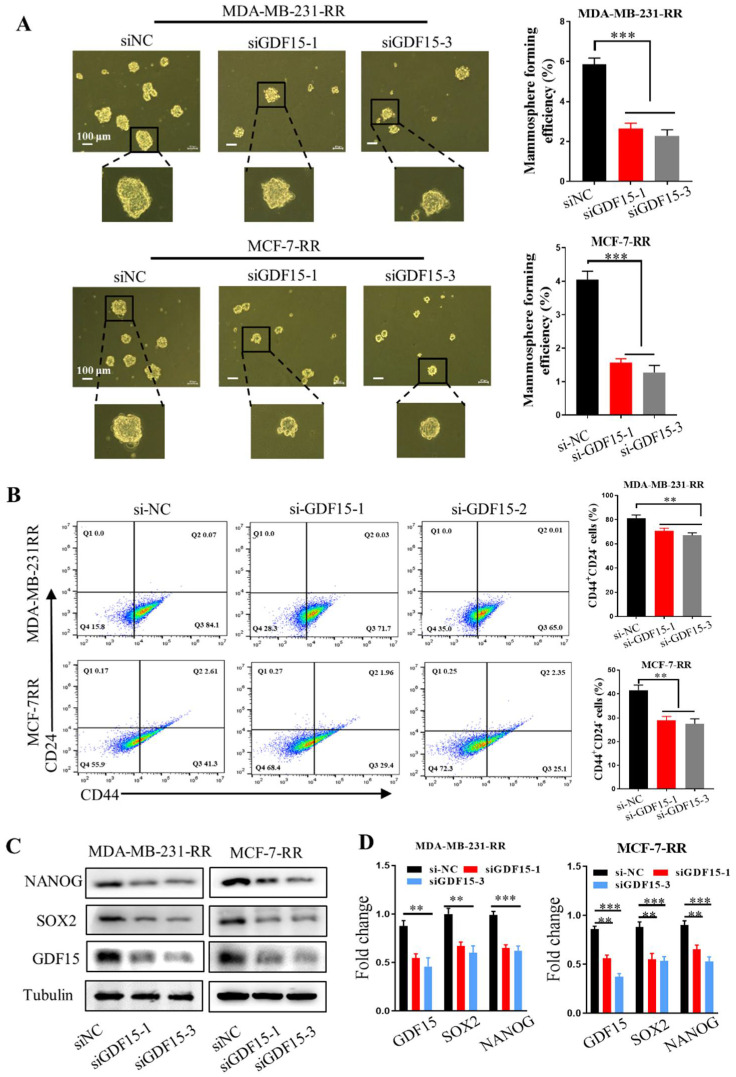
GDF15 knockdown attenuated the stem-like traits of MDA-MB-231-RR and MCF-7-RR cells. (**A**) The stemness of MDA-MB-231-RR and MCF-7-RR cells was evaluated by the mammosphere formation assay after treatment with siGDF15. (**B**) Flow cytometry assay of CD44^+^ CD24^−^ cells in the radioresistant cells transferred with siGDF15 by using PE-CD24 and FITC-CD44 antibodies. (**C**,**D**) Western blot assay of the expressions of stemness-associated markers (SOX2, NANOG) in the radioresistant cells transferred with siGDF15. *** *p* < 0.001, ** *p* < 0.01.

Considering the EMT process endows the mesenchymal properties of cancer cells and reflects the ability of cancer cells to enter CSC status [32,33], we validated the function of GDF15 in maintaining the stemness of radioresistant breast cancer cells. Mammosphere formation assay showed that GDF15 silencing significantly impaired the sphere formation capacities of radioresistant cells (Figure 6A). Moreover, knockdown of GDF15 also reduced the number of stem-like CD44^+^ CD24^−^ subpopulation in the MDA-MB-231-RR and MCF-7-RR cells (Figure 6B) and suppressed the expressions of stemness-associated proteins of NANOG and SOX2 in these cells (Figure 6C,D). Therefore, GDF15 mediated the radioresistance of breast cancer by maintaining the stemness of cells.

## 3. Discussion

Ample evidence has demonstrated that radiotherapy can markedly mitigate the postoperative recurrence and improve the survival of patients with breast cancer, colon cancer, prostate cancer and esophagus cancer [34]. Radiotherapy is even deemed to be a standard procedure after breast-preserving surgery. Compared with non-radiotherapy postoperative patients, adjuvant radiotherapy effectively abates the risk of recurrence [35]. However, there was still a group of patients with a short survival period due to the tumor radioresistance [36]. Therefore, understanding prognosis-related biomarkers and pleiotropic mechanisms has great significance in improving the prognosis of breast cancer patients.

Based on the datasets GSE59732 and GSE59733 in the GEO database, we screened out five common DEGs (GDF15, IFIT1, CDKN1A, FAS, and BTG2). By employing the TCGA database analysis of these genes, we validated that the GDF15 expression was strongly associated with the poor prognosis of 276 breast cancer patients after radiotherapy. The expression of GDF15 was related to the tumor types, histologic subtypes, nodal metastasis status, and individual cancer stages. In the past two decades, accumulated evidence has demonstrated that GDF15 is a stress-induced cytokine and could be up-regulated in the context of several diseases, including heart, kidney, liver and lung [37,38,39]. However, most of the previous studies mainly focused on the function of GDF15 in obesity and cardiac metabolic diseases [40,41,42], and little is known about how GDF15 affects the radioresistance of cancer.

This study revealed that GDF15 was associated with radioresistance of breast cancer for the first time. It was found that GDF15 was significantly up-regulated in the irradiated breast cancer cells and highly expressed in the stable radioresistant cell lines MDA-MB-231-RR and MCF-7-RR cells. Although several signaling pathways related to DNA damage response (such as p53, ATM, ATR signaling pathways) were enriched through bioinformatics analysis, and many articles have reported the relationship between GDF15 and these pathways [43,44,45], we desire to explore other new signal pathways that may be involved in the role of GDF15 protein. It was reported that the occurrence of the EMT process and the generation of stem cell population was the main reason for cancer radioresistance [30,46]. Our experiments also demonstrated that these radioresistant breast cancer cells had enhanced EMT properties and stem-like traits. When GDF15 was silenced, the EMT characteristics of MDA-MB-231-RR and MCF-7-RR cells were weakened, i.e., the migration and invasion abilities were significantly inhibited together with the down-regulation of snail, slug, and vimentin protein expressions and up-regulation of E-cadherin expression. Furthermore, knockdown of GDF15 also hindered the formation of mammospheres and reduced the proportion of CD44^+^ CD24^−^ cells and the expression of NANOG and SOX2 associated with the stemness of cells in the population of radioresistant cells.

An increasing number of studies indicated that CSCs might be quite plastic and closely linked to EMT, and the enhancement of EMT and stem-like traits increased the resistance of patients to irradiation [47,48]. TGF-β is a well-studied strong inducer for EMT, which can generate dynamic cytoskeletal remodeling and morphological change of the epithelial to mesenchymal in the cancer cells and can promote radioresistance of cancer by regulating EMT [47,49,50]. However, as a member of TGF-β superfamily, there are still few reports on the role of GDF15 in tumor radioresistance, but more studies about the roles of GDF15 focus on its adverse effect on cancer cachexia [51], the regulation of immune microenvironments [52,53], and diagnostic marker of multiple solid tumors [54,55,56]. Therefore, exploring the effect of GDF15 on the radioresistance of breast cancer may open up a new avenue for clinical radiotherapy. The discovery that GDF15 affects the radiosensitivity of breast cancer by regulating the EMT and stemness of radioresistant cells provides us tangible proof to propose that GDF15 can be used as a molecular target for the combination treatment of GDF15 inhibitor and radiotherapy, although there is a possibility that RT kills non-EMT/stem cells and enriched EMT/CSC, leading to increased GDF15 expression. We will further explore the specific regulatory mechanism of GDF15 in radioresistant breast cancer stem cells in the future.

In conclusion, this study has brought about a novel prognostic indicator, GDF15, in predicting the survival of breast cancer patients post-radiotherapy with bioinformatic analysis and in vitro confirmation. Mechanically, GDF15 contributed to the radioresistance of breast cancer cells by enhancing the EMT process and stem-like traits. All referred evidence prompts that GDF15 is a promising radiotherapeutic target for breast cancer patients and targeting GDF15 may provide a new strategy for improving the radiotherapy efficiency of breast cancer patients.

## 4. Materials and Methods

### 4.1. Microarray Datasets

Two microarray datasets of GSE59732 (Affymetrix GPL571 platform, Affymetrix Human Genome U133A 2.0 Array) and GSE59733 (GPL18990 platform, Affymetrix Human Almac Xcel Array) were obtained from the GEO database. The samples in the MCF7 cell line (GSM1444569–GSM1444574) and ZR751 cell line (GSM1444563–GSM1444568) under the treatment of 0 and 5 Gy were employed for the analysis of DEGs were chosen from the GSE59732 dataset. The dataset GSE59733 contained gene expression profiles of the paired tumor samples from 18 breast cancer patients pre-radiotherapy (GM1444655–GSM1444657, GSM1444659–GSM1444664) and post-radiotherapy (GSM1444653, GSM1444654, GSM1444658, GSM1444666, GSM1444668–GSM1444671).

### 4.2. Data Preprocessing in GSE59732

To screen the DEGs, R/Bioconductor software 3.6.1 (Auckland, New Zealand) was used to analyze the GSE59732 dataset. The edgeR package was applied to import, organize, filter and normalize the data, and the Limma package was applied to perform the DEGs analysis and gene set testing with the voom method, linear modeling and empirical Bayes moderation. *p* < 0.05 and |log2 Fold Change  FC)| > 1 were set as the standard to extract appropriate genes. The pheatmap and ggplot2 packages were subsequently applied to develop the heatmap and volcano plot for the visualization of DEGs. In order to predict the enriched pathway of DEGs in MCF-7 and ZR751 cell lines from GSE59732, we performed GO enrichment analysis and KEGG pathway analysis with the Cluster Profiler package in the R software (Bioconductor).

### 4.3. Data Preprocessing in GSE59733

The DEGs between breast tumor samples pre- and post-radiotherapy in the GSE59733 dataset were screened out using GEO2R online analysis tool. GEO2R is an interactive web tool that mainly performs the analysis of chip data with the help of R and Limma packages. *p* < 0.05 and |log2 Fold Change FC)| > 1 were defined as thresholds.

### 4.4. Analyses of Function Enrichment and Pathway Network

Intersecting genes between GSE59732 and GSE59733 datasets were performed using the VENNY online analysis tool (http://bioinfogp.cnb.csic.es/tools/venny, accessed on 15 April 2020), and the result was presented in a Venn diagram. The protein–protein interaction (PPI) network among DEGs was constructed using the database STRING (version 11.0, ELIXIR, Hinxton, Cambridgeshire, UK). Significant functional pathways were recruited in the overlapped genes by means of the FunRich software (Bundoora, Australia).

### 4.5. Clinical Data Analysis

The Kaplan–Meier estimation of overall survival and the co-expression analysis of GDF15 and CDKN1A in breast cancer patients with radiotherapy were performed using the online tool LinkedOmics (http://www.linkedomics.org, accessed on 15 April 2020). The correlation analysis between GDF15 and CDKN1A in breast cancer was performed using the GEPIA database (http://gepia.cancer-pku.cn, accessed on 15 April 2020). The online tool UALCAN (http://ualcan.path.uab.edu, accessed on 15 April 2020) was then used to carry out the analysis between GDF15 expression and cancer types, histologic subtypes, nodal metastasis status and individual cancer stages of breast cancer in the TCGA database.

### 4.6. Cell Culture and Irradiation

Human breast cancer cell lines of MCF-7, T-47D, MDA-MB-231 and MDA-MB-468 were obtained from Shanghai Cell Bank (Chinese Academy of Science, Shanghai, China). All cell culture reagents were purchased from GIBCO (Invitrogen, Grand Island, NY, USA). MCF-7 and MDA-MB-231 cells were cultured in Dulbecco’s modified Eagle’s medium (DMEM) supplemented with 10% fetal bovine serum (FBS) and 1% penicillin/streptomycin. T-47D and MDA-MB-468 cells were cultured in RPMI-1640 medium containing 10% FBS and 1% penicillin/streptomycin. All cell lines were maintained in a humidified atmosphere containing 5% CO_2_ at 37 °C. Cells in the logarithmic phase were irradiated with 4 Gy X-rays by using the X-ray Irradiator (X-RAD 320, PXI, Madison, CT, USA) at a dose rate of 1 Gy/min. After 12 h of irradiation, cells were collected for further assays.

### 4.7. Establishment of Radioresistant Breast Cancer Cell Lines

MDA-MB-231 and MCF-7 cells were irradiated with X-rays at a dose rate of 1 Gy/min at 320 kV and 12.5 mA. MDA-MB-231 received a total dose of 50 Gy (cells were irradiated with 4 or 6 Gy once weekly followed by a recovery period of 1~2 weeks). MCF-7 cells received a total dose of 60 Gy (cells were irradiated with 4 Gy twice weekly, followed by a recovery period of 1~2 weeks). The radioresistant cells (MDA-MB-231-RR and MCF-7-RR) were derived from surviving clones after fractionated irradiation.

### 4.8. Clonogenic Survival Assay

The acquisition of radioresistance among different cell lines were confirmed by clonogenic survival assay. Cells in each group were collected, and the appropriate number of cells were seeded in 6-well plates. After 24 h, cells were exposed to 0, 2, 4, 6, and 8 Gy of X-rays, and the number of inoculated cells was 200, 200, 400, 600, and 800 cells/well, respectively. Then the cells were cultured for another 10~15 days in an incubator containing 5% CO_2_ at 37 °C. Subsequently, the colonies were washed with PBS twice and fixed with 10% formalin for 10 min, stained with 0.1% crystal violet for 10 min, and counted under a microscope. More than 50 cells were considered as an effective colony. Cell survival fraction (SF) was calculated according to the equation: SF = PE_n Gy_/PE_0 Gy_ (PE: plating efficiency, PE = colonies counted/cells seeded). PE_n Gy_ represents the PE of irradiated cells, PE_0 Gy_ Represents the PE of unirradiated cells. Graphpad prism 8.0 software (San Diego, CA, USA) was used to calculate the SF of each group. A survival curve was stimulated by the multi-target single hit model: SF = 1 − (1 − e^−D/D0^)^N^. D_0_ is the radiation dose received when about 37% of the cells survive. The sensitization enhancement ratio (SER) is calculated as D_0_ (control)/D_0_ (treatment).

### 4.9. Western Blotting Assay

Total proteins were extracted from culture cells with pre-cold SDS lysis buffer containing protease inhibitor (phenylmethanesulfonyl fluoride (PMSF) (Beyotime Biotech., Haimen, Jiangsu, China). BCA Protein Assay Kit (Beyotime) was used to determine the concentration of proteins, and the loading amount was calculated by the concentration of the obtained protein sample, where 30 μg proteins were loaded in each lane. The protein lysates were separated in 12.5% resolving gel and transferred to polyvinylidene difluoride (PVDF) membrane (Millipore Corp., Billerica, MA USA). After blocking with 5% skimmed milk for 2 h at room temperature, the membrane was incubated with the primary antibodies against GDF15, SOX2, NANOG (Proteintech, Wuhan, China), slug, snail, vimentin, E-cadherin, (Cell Signaling Technology, Beverly, MA, USA), Tublin (Beyotime) overnight at 4 °C, the dilution ratio of all used primary antibodies was 1:1000. The superfluous primary antibody was washed away with TBST buffer triply for 10 min each time at room temperature. The membrane was then incubated with goat anti-rabbit IgG-HRP secondary antibody (1:5000, Beyotime) for 2 h at room temperature, and the excess secondary antibody was washed triply with TBST buffer for 10 min each time at room temperature. After that, the chemiluminescence detection was performed with the ChemiDoc^TM^ XRS imager (Bio-Rad, Hercules, CA, USA).

### 4.10. RNA Extraction and PCR Assay

Total RNA was extracted from MCF-7, T-47D, MDA-MB-231 and MDA-MB-468 cells using Trizol (Invitrogen Life Technologies, Carlsbad, CA, USA) following the manufacturer’s protocol. Briefly, 1 × 10^6^ cells were added to 500 μL Trizol agent, centrifuged at 12,000 rpm at 4 °C for 5 min to remove insoluble matter. After chloroform extraction and precipitation with isopropanol, RNA was washed twice with 75% ethanol and then dissolved in 15 μL of RNase-free water. The concentration and A260/A280 of RNA samples were measured using the NanoDrop spectrophotometer. The cDNA synthesis was performed using FastKing gDNA Dispelling RT SuperMix kit (Tiangen, Beijing, China). The sequences of primers are as following: GDF15 (forward primer, 5′-TCA CGC CAG AAG TGC GGC TG-3′, reverse primer, 5′-GGA CAG TGG TCC CCG TTG CG-3′) and β-actin (forward primer, 5′-GGG ACC TGA CTG ACT ACC TC-3′, reverse primer, 5′-TCA TAC TCC TGC TTG CTG AT-3′). RT-PCR products were analyzed with 1.5% agarose gel electrophoresis and stained with GelRed (Biotium Inc., Fremont, CA, USA) for visualization under ultraviolet light. The mRNA expression levels of DEGs in breast cancer cells were quantified by quantitative real-time PCR (qRT-PCR) with the SuperReal PreMix plus SYBR Green kit (Tiangen, Beijing, China) using an Mx3000P instrument (Agilent, Santa Clara, CA, USA). β-actin was used to normalize the expression value of DEGs as the endogenous reference gene.

### 4.11. RNA Interference

GDF15-targeting siRNAs for mouse cells (si-GDF15-1, si-GDF15-2, si-GDF15-3) and its negative control (si-NC) were designed and synthesized by Ribobio (Guangzhou, China). MDA-MB-231RR and MCF-7RR cells (1.0 × 10^5^ per well) were seeded into 6-well plates, after 20 h, cells were cultured in fresh medium supplemented with 2% FBS and transfected with 50 nM si-GDF15 or its scramble control using riboFECTTM CP Reagent (Ribobio, Guangzhou, China) according to the manufacturer’s instruction. After 48 h, the siRNA transfection efficiency was identified by Western blot assay.

### 4.12. Transwell Invasion Assay

The invasive ability of MDA-MB-231 and MCF-7 cells was determined by transwell invasion assay. The bottom of 24-well chemotaxis chamber (Corning Inc., Cornyn, NY, USA) was filled with complete DMEM medium and the upper chambers were coated with the mixture of serum-free medium and matrigel (6:1; BD Biosciences, Franklin Lake, NJ, USA). The cells were digested and washed twice with PBS, then resuspended with 200 μL serum-free medium and added into the upper chambers, then cultured in a humidified atmosphere containing 5% CO_2_ at 37 °C. After 48 h of incubation, the matrigel on the upper chamber was removed and the membrane was fixed with 10% formalin for 10 min, stained with 0.1% crystal violet for 10 min at room temperature. Cells were photographed and counted under a microscope. Five fields were randomly selected to count the number of cells in each field.

### 4.13. Wound Healing Assay

MDA-MB-231 and MCF-7 cells (1 × 10^5^) were seeded in a 6-well plate and scraped in a straight line to create a “scratch” with a 200 μL pipette tip when cells were grown to nearly 90% confluence. Cells were washed with PBS gently to remove the loose cells, and then cultured in serum-free DMEM medium. Cell images were acquired with a microscope after 0 and 48 h of cell culture and the wound widths were measured by Image-ProPlus software (Media Cybernetics Inc., Rockville, MD, USA). The migration rate is the ratio of the migrated distance to the wound width at 0 h of cell culture.

### 4.14. Mammosphere Formation

The ability of tumor sphere formation is an important method for the identification of cancer stem cells in vitro, mainly to determine the self-renewal capability of individual cells in suitable conditioned media, which is generally indicated by the cell spheroid formation efficiency (SFE). This method is based on the growth characteristics of tumor stem cells, that is, under the serum-free and non-adherent culture conditions, the differentiated tumor cells will die, while the stem-like tumor cells will survive and proliferate to form three-dimensional cell spheres suspended in the medium. In brief, MDA-MB-231 and MCF-7 cells were digested into single cell suspension and seeded in ultralow attachment plates (Corning, 4000 cells/well) with DMEM/F12 medium containing 1 × B27 (Gibco), 1 × N2 (Gibco), bFGF (25 ng/mL), and E GF (20 ng/mL) and cultured at 37 °C with 5% CO_2_ for 10 days. Spheres greater than 50 μm diameter were counted and photographed using a microscope. Mammosphere formation efficiency (MFE) was calculated as MFE (%) = (the number of mammospheres identified per well/the number of cells seeded per well) × 100%.

### 4.15. Flow Cytometry Assay of CD24 and CD44

After treatment, the cells were digested and washed twice with cold PBS containing 2% BSA, then resuspended in 100 μL PBS and incubated with the antibody FITC-IgG, PE-IgG and FITC-CD44, PE-CD24 (BioLegend, San Diego, CA, USA) for 30 min at 4 °C in the dark. The expressions of stemness-related markers of CD24 and CD44 were detected by flow cytometry, and the ratio of CD44^+^ and CD24^−^ cells was analyzed.

### 4.16. Statistical Analysis

The experimental data from at least three independent experiments are presented as the mean ± SD by using GraphPad Prism 8.0 software (San Diego, CA, USA). A Student’s t-test was performed to compare the difference of independent groups, and the significant level was considered at *p* < 0.05. 

## Data Availability

The data presented in this study are available on request from the corresponding author upon reasonable request.

## Supplementary Materials

The following supporting information can be downloaded at: https://www.mdpi.com/article/10.3390/ijms231810911/s1.
