# Supplementary material for: GDF15 Contributes to Radioresistance by Mediating the EMT and Stemness of Breast Cancer Cells
Source: Int J Mol Sci. 2022 Sep 18;23(18):10911. doi: 10.3390/ijms231810911 (PMC9504016; doi:10.3390/ijms231810911)
Supplement: Supplementary file 1 [file ijms-23-10911-s001.zip › ijms-1810199-supplementary.pdf]

Supplementary Tables

Table S1. Top 10 up-regulated and down-regulated DEGs between irradiated and non-irradiated MCF-7 cells from GSE59732 dataset.

Gene	Average expression value	Log2 FC	P-value	Expression alternation
FAS	7.581564	1.882813	8.05E-05	Up
UGT2B15	7.099253	1.646847	2.95E-05	Up
GADD45A	9.476228	1.530667	7.00E-06	Up
GDF15	10.387403	1.394882	9.93E-06	Up
IFIT1	9.656978	1.283209	4.48E-05	Up
ACTA2	7.071137	1.279755	1.42E-03	Up
ATF3	6.62637	1.266457	3.80E-05	Up
CDKN1A	8.279434	1.155655	5.45E-04	Up
BTG2	8.809003	1.14495	8.82E-05	Up
SLC1A1	6.032318	1.107265	5.63E-04	Up
DTL	7.757903	-1.429694	5.76E-05	Down
HELLS	8.133293	-1.352927	4.86E-05	Down
CDC45	7.778412	-1.227417	5.86E-05	Down
RRM2	11.310513	-1.196906	2.76E-05	Down
CENPI	6.880251	-1.160084	3.09E-04	Down
CDT1	8.195456	-1.142144	1.30E-05	Down
BMP5	8.582497	-1.140018	1.16E-04	Down
MCM10	8.730336	-1.139252	4.29E-05	Down
CDC6	7.514279	-1.106088	7.61E-05	Down
E2F8	8.486154	-1.063678	7.12E-05	Down

Table S2. Top 10 up-regulated and down-regulated DEGs between irradiated and non-irradiated ZR-751 cells from GSE59732 dataset.

Gene	Average expression value	Log2 FC	P-value	Expression alternation
GDF15	9.430798	2.254643	1.14E-07	Up
ACTA2	7.882679	2.178834	3.13E-07	Up
BTG2	8.487721	2.169643	2.27E-07	Up
FAS	7.421035	1.886192	2.23E-06	Up
CDKN1A	11.113523	1.82961	3.10E-07	Up
HIST2H2BE	9.493893	1.648001	1.28E-06	Up
FOS	5.920792	1.595922	9.00E-06	Up
ZMAT3	7.039384	1.544735	9.19E-07	Up
SLC16A4	6.687988	1.520954	1.97E-05	Up
FDXR	7.874514	1.479282	1.25E-05	Up
ASPM	8.175642	-3.127638	1.06E-08	Down
NDC80	8.262077	-2.914874	2.77E-08	Down
AURKB	8.017115	-2.600507	3.64E-08	Down
BUB1	7.921686	-2.789411	4.78E-08	Down
ESPL1	8.569054	-2.459281	5.21E-08	Down
CDCA3	7.759605	-2.573204	6.43E-08	Down
MKI67	8.5235	-2.296088	7.05E-08	Down
PIMREG	8.030846	-2.503399	8.76E-08	Down
MCM10	9.071397	-2.423183	1.11E-07	Down
DTL	8.587437	-2.17639	1.20E-07	Down

Table S3. Top 10 up-regulated and down-regulated DEGs in breast cancer patients pre- and post-radiotherapy.

Gene	Log2 FC	P-value	Expression alternation
NR4A1	3.408058	5.98E-06	Up
FOS	3.359085	4.98E-06	Up
DUSP1	3.177125	1.10E-06	Up
CDKN1A	2.920849	1.08E-07	Up
CLCA2	2.812179	4.45E-05	Up
KRT6B	2.744143	1.13E-03	Up
KRT5	2.718304	1.02E-03	Up
SLC2A3	2.651352	1.88E-07	Up
EGR1	2.522909	4.46E-04	Up
GDF15	2.493734	4.95E-05	Up
CLEC2D	-1.57892	5.22E-03	Down
TTC34	-1.57797	3.80E-03	Down
MUC12	-1.42348	2.09E-04	Down
PPP4R2	-1.40474	2.55E-03	Down
FGFR3	-1.39588	3.66E-04	Down
C1orf64	-1.38576	9.57E-03	Down
ACTR3C	-1.26667	7.96E-03	Down
FLJ45340	-1.26638	9.36E-03	Down
ZNF595	-1.25785	2.62E-02	Down
CCNB1	-1.23352	2.81E-02	Down
